# Pleural fluid neutrophil extracellular traps are associated with disease severity and risk of 1-year mortality in pleural infection: an observational, international, multicohort study (TORPIDS-3)

**DOI:** 10.1183/13993003.00113-2026

**Published:** 2026-08-27

**Authors:** Julia Y. Chu, Samuel Hollins, Alguili Elsheikh, Stephen Gerry, Nikita Manoharan, Tryfon Zarganes-Tzitzikas, Yinzhou Feng, Elie Antoun, Eihab O. Bedawi, John P. Corcoran, Amy Gilmour, Federico Mei, Federica Monaco, Marco Tomasetti, Argyrios Tzouvelekis, Electra Koulousousa, Fotios Sampsonas, Malvika Bhatnagar, Ellice Z. Chen, Paul E. Brennan, Tao Dong, Ian D. Pavord, Yuanlin Song, James D. Chalmers, Julian C. Knight, Najib M. Rahman, Nikolaos I. Kanellakis

**Affiliations:** 1 CAMS Oxford Institute, Chinese Academy of Medical Sciences and Peking Union Medical College, University of Oxford, Oxford, UK; 2 Laboratory of Respiratory Translational Research, Nuffield Department of Medicine, University of Oxford, Oxford, UK; 3 Oxford Centre for Respiratory Medicine, Churchill Hospital, Oxford University Hospitals NHS Foundation Trust, Oxford, UK; 4 Centre for Statistics in Medicine, Nuffield Department of Orthopaedics, Rheumatology and Musculoskeletal Sciences, University of Oxford, Oxford, UK; 5 Centre for Medicines Discovery, Nuffield Department of Medicine, University of Oxford, Oxford, UK; 6 Alzheimer's Research UK Oxford Drug Discovery Institute, Centre for Medicines Discovery, Nuffield Department of Medicine, University of Oxford, Oxford, UK; 7 Centre for Translational Immunology, Nuffield Department of Medicine, University of Oxford, Oxford, UK; 8 Division of Molecular and Clinical Medicine, University of Dundee, Ninewells Hospital and Medical School, Dundee, UK; 9 Respiratory Disease Unit, Department of Internal Medicine, University Hospital, Ancona, Italy; 10 Department of Biomedical Sciences and Public Health, Polytechnic University of Marche, Ancona, Italy; 11 Occupational Medicine, Department of Clinical and Molecular Sciences, Polytechnic University of Marche, Ancona, Italy; 12 Respiratory Department, Patras University Hospital, University of Patras, Patras, Greece; 13 Pulmonary Critical Care and Sleep Medicine Department, Yale School of Medicine, New Haven, CT, USA; 14 MRC Translational Immune Discovery Unit, MRC Weatherall Institute of Molecular Medicine, Radcliffe Department of Medicine, University of Oxford, Oxford, UK; 15 Respiratory Medicine Unit, Oxford NIHR Biomedical Research Centre, Nuffield Department of Medicine, University of Oxford, Oxford, UK; 16 Shanghai Key Laboratory of Lung Inflammation and Injury, Shanghai Respiratory Research Institute, Department of Pulmonary Medicine, Zhongshan Hospital, Fudan University, Shanghai, China; 17 Radcliffe Department of Medicine, University of Oxford, Oxford, UK; 18 Centre for Human Genetics, Nuffield Department of Medicine, University of Oxford, Oxford, UK; 19 National Institute for Health Research Oxford Biomedical Research Centre, University of Oxford, Oxford, UK; 20 These authors contributed equally

## Abstract

**Background:**

Influx of large numbers of neutrophils is characteristic of pleural infection; however, neutrophil behaviour is understudied. We designed an observational, multicohort study with the aims to discover and validate the association between neutrophil extracellular traps (NETs), disease severity, 1-year survival and the extent of sonographic septations.

**Methods:**

We analysed five independent datasets across three countries. The PILOT cohort (UK, n=215) was used as a discovery cohort for the association of NETs with disease severity, with validation in four independent cohorts across three countries (UK, Greece and Italy, n=100). The PILOT cohort was further analysed to assess the association of NETs with 1-year survival and the development and extent of sonographic septations. A separate cohort (Oxford-Osler, UK, n=30) was used to assess the relationship between NETs and the need for intrapleural enzyme therapy as rescue treatment.

**Results:**

A 10-unit increase in NETs was associated with 13% higher odds of being in a higher RAPID score group (OR 1.13, 95% CI 1.02–1.27; p=0.02), and this association remained robust in the independent validation cohort (OR 1.29, 95% CI 1.11–1.40; p=0.0005). A 10-unit increase in NETs was also associated with a 15% higher risk of 1-year mortality (HR 1.15, 95% CI 1.01–1.32; p=0.04) independent of the RAPID score, and a 17% higher likelihood of sonographic septations (OR 1.17, 95% CI 1.01–1.37; p=0.04). Citrullinated fibrin was associated with septation severity (OR 1.10, 95% CI 1.04–1.16; p=0.00005).

**Conclusions:**

Pleural fluid NETs are a biomarker of disease severity and 1-year mortality, in pleural infection. Future studies are required to assess the combination of NET-targeting strategies in combination with existing treatments.

## Shareable abstract

**Pleural fluid neutrophil extracellular traps are associated with disease severity and risk of 1-year mortality in pleural infection** https://bit.ly/4tFbz0Z

## Graphical abstract

**Figure:**
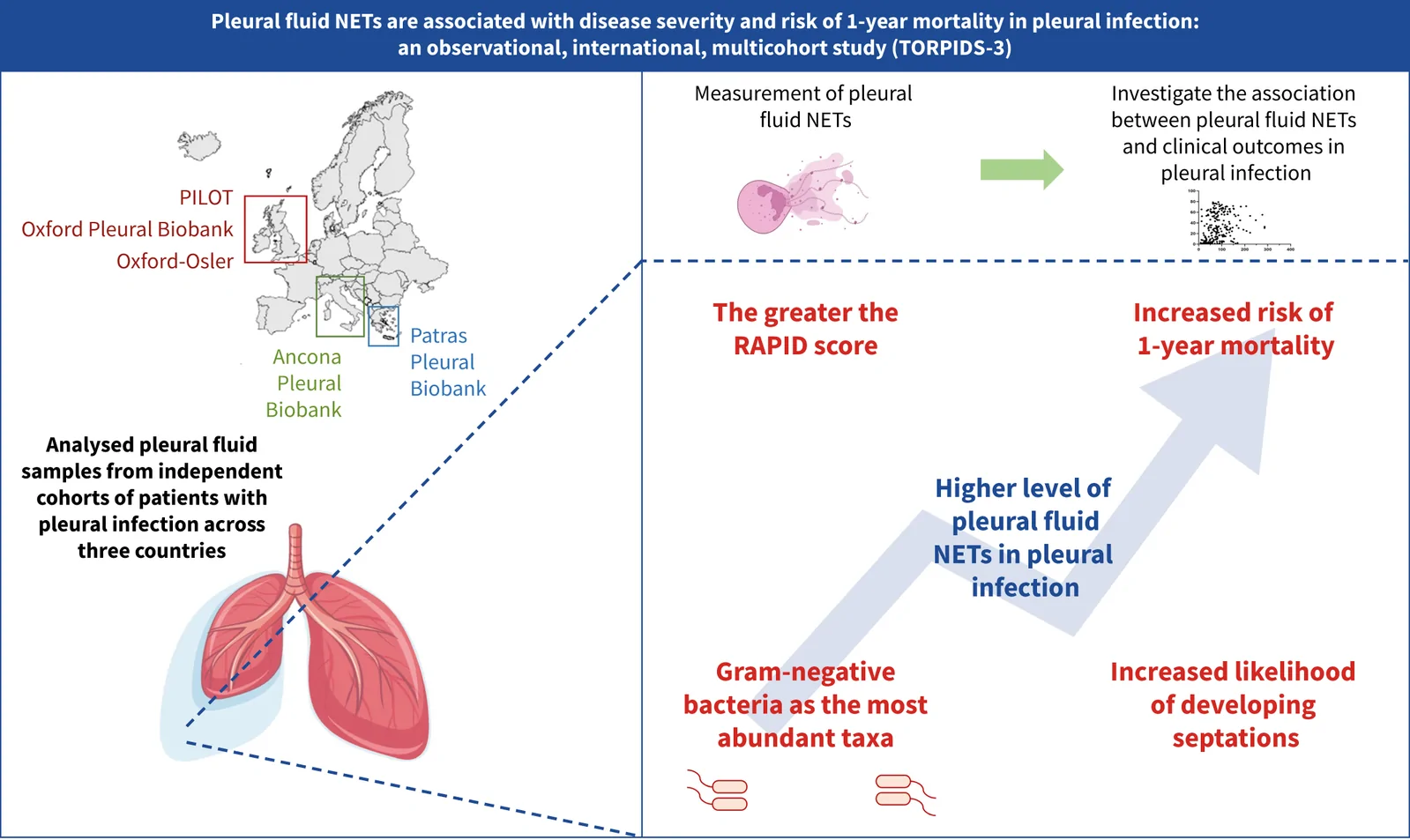
Overview of the study. NETs: neutrophil extracellular traps.

## Introduction

Pleural infection is a severe disease bearing significant morbidity and mortality worldwide [1, 2]. Patients require prolonged hospitalisation and invasive treatments of variable efficacy, contributing to discomfort and uncertainty, while increasing the costs for the healthcare service [3]. Clinical outcomes for patients with pleural infection remain poor, reflecting our incomplete understanding of the disease's underlying biology. Despite the use of antibiotics and intrapleural enzyme therapy (IET), the mean 3-month mortality remains ∼10%, with 1-year mortality ∼20%, increasing to 35% in the elderly or immunocompromised [4–6].

Neutrophils are innate cell phagocytes which play a central role in the immune response to bacterial infections. We have shown that pleural infection patients exhibit diverse levels of intrapleural neutrophil-mediated activity which are associated with microbiology [7].

Neutrophils extrude neutrophil extracellular traps (NETs), an antimicrobial defence mechanism that has been recognised as a key driver of inflammatory conditions. Dysregulated NETosis promotes immunothrombosis leading to tissue damage and contributing to pathology [8]. Elevated levels of NETs have been associated with the severity of acute and chronic conditions, including COVID-19, bacterial pneumonia, sepsis and bronchiectasis [8–11]. These discoveries have led to the development and clinical use of drugs that target NET-related components in bronchiectasis [12].

A better understanding of the biological role, function and effect of NETs in pleural infection may inform therapeutic approaches and lead to improved clinical outcomes. Here we present the results of an observational, multicohort study to investigate the association between pleural fluid NETs with disease severity, 1-year survival, and the development and severity of sonographic septations.

## Methods

### Study design

The present multicohort study analysed pleural fluid specimens and clinical data from five independent cohorts across three countries with the aim to investigate the biological role of NETs in pleural infection (table 1).

**TABLE 1:** Demographics for the five pleural infection cohorts

	PILOT		Oxford Pleural Biobank		Oxford-Osler		Patras Pleural Biobank		Ancona Pleural Biobank	
		Missing values		Missing values		Missing values		Missing values		Missing values
**Age (years)**	59.5±16.9	6 (3)	56.2±18.5	0 (0)	64.1±16.1	0 (0)	64.7±14.3	0 (0)	66.7±18.1	0 (0)
**Sex**		6 (3)		0 (0)		0 (0)		0 (0)		0 (0)
Female	53 (23)		12 (26)		11 (31)		2 (18)		3 (23)	
Male	177 (74)		34 (74)		24 (69)		9 (82)		10 (77)	
**WBC count (×10^9^ L^−1^)**	15.97±14.28	7 (3)	13.95±5.02	1 (2)	16.07±8.54	0 (0)	16.35±7.91	0 (0)	15.37±7.63	0 (0)
**CRP (mg·L^−1^)**	249.24±520.54	20 (9)	181.10±92.43	2 (4)	209.93±127.02	1 (3)	178.73±128.85	0 (0)	230.16±103.27	0 (0)
**Pleural fluid LDH (IU·L^−1^)**	6580.18±14 497.96	54 (25)	7576.47±14 175.8	9 (22)	5475.15±10 772.03	8 (23)	1216.55±1210.78	0 (0)	1002.25±965.64	0 (0)
**RAPID score**		6 (3)		0 (0)		0 (0)		0 (0)		0 (0)
Low	88 (39)		23 (50)		10 (29)		2 (18)		1 (8)	
Medium	104 (46)		19 (41)		13 (37)		7 (64)		7 (54)	
High	28 (12)		4 (9)		12 (34)		2 (18)		5 (38)	
**Infection source**		6 (3)		0 (0)		0 (0)		0 (0)		0 (0)
Community	220 (91)		43 (93)		35 (100)		11 (100)		13 (100)	
Hospital	15 (6)		3 (7)		0 (0)		0 (0)		0 (0)	

Data are presented as mean±sd or n (%). WBC: white blood cell; CRP: C-reactive protein; LDH: lactate dehydrogenase.

#### Pleural infection samples

In all cohorts adult patients were assessed by the recruiting physician for evidence of infection, on the basis of fever, elevated peripheral white blood cell count or elevated serum inflammatory markers (C-reactive protein), and diagnosed with pleural infection on the basis of standard-of-care guidelines [2]. For all cohorts patients were recruited under the same clinical criteria. Detailed clinical inclusion and exclusion criteria are described in the supplementary material.

#### Negative control samples

Pleural fluid samples from patients with no evidence of infection and confirmed diagnosis of malignant pleural effusion, heart failure or non-specific pleuritis were used as negative controls (supplementary table S1).

All donors provided written informed consent, and specimens were collected prospectively for all cohorts.

### Cohorts

The PILOT [13] cohort (UK, n=215; Medical Sciences Interdivisional Research Ethics Committee: IDREC R74885/RE001) was studied to investigate the association between NETs, microbiology, sonographic septations and risk of 1-year mortality.

Disease severity was assessed using the RAPID score [14]. The association between NETs and disease severity was evaluated in the PILOT cohort and validated in an external cohort consisting of Oxford Pleural Biobank (UK, n=46; Oxford Centre for Histopathology Research (OCHRe): 22/A093), Oxford-Osler (UK, n=30; OCHRe: 25A037), Patras Pleural Biobank (Greece, n=11; Patras University Hospital Research Ethics Committee: 170/14.03.2024) and Ancona Pleural Biobank (Italy, n=13; Marche Region Ethical Committee: 2023/396/8033).

The trial (TORPIDS-3) is registered with ClinicalTrials.gov (NCT07194915).

### Sample processing

All samples were processed on the day of collection to preserve their integrity, as previously described [15]. Harvested supernatants were kept at −80°C until use in assays.

### Molecular microbiology

The microbiological profiling of the PILOT cohort was previously performed (TORPIDS study) using 16S rRNA next-generation sequencing [15].

### NETs complex measurements

NETs were measured as previously described [11, 16]. In brief, anti-ELANE (neutrophil elastase) antibody (sc-55549; Insight Biotechnology, London, UK) was coated onto a 96-well plate and incubated overnight at 4°C. After three washes with DPBS, 20 µL pleural fluid samples was added to wells in conjunction with 80 µL incubation buffer containing a peroxidase-conjugated anti-DNA antibody (a component of the Cell Death ELISA kit, 11544615001; Roche, Basel, Switzerland; dilution 1:25) and the plate was incubated for 2 h at room temperature with gentle shaking at 300 rpm. After three washes, 100 µL TMB solution was added to the wells and the plate was incubated for 20 min at room temperature with protection from light. To stop the reaction, 50 µL stop solution was added to the wells. Absorbance at 405 nm was measured using a microplate reader. The median (interquartile range) coefficient of variation across triplicate measurements was 6.12% (2.35–12.85%).

### Statistical analysis

Statistical analysis and plotting of graphs were performed in R version 4.5.1 (www.r-project.org) and GraphPad Prism version 10.6 (GraphPad, San Diego, CA, USA).

The Mann–Whitney U-test was used to compare differences between two groups. Differences between three or more groups were evaluated by the Kruskal–Wallis test followed by pairwise Wilcoxon rank-sum tests with Holm adjustment to control for family-wise error rate. Ordinal logistic regression was used to assess the association between ELANE NETs and the RAPID score and sonographic septations, and to evaluate the relationship between citrullinated fibrin and sonographic septations. For this the polr function from the MASS R package version 7.3.65 was used. The proportional odds assumption was tested using the Brant test (Brant R package version 0.3.0) and was found to hold for all tests (p>0.05). Univariate and multivariate Cox hazard regression models were performed to assess the association between pleural fluid NETs and 1-year survival. Logistic regression models were applied to examine the association between NETs and purulence and the requirement for IET. Model estimates are reported as odds ratio, and p-values were calculated using the normal approximation. A Friedman test followed by paired Wilcoxon signed-rank tests with Benjamini–Hochberg (false discovery rate) correction for multiple comparisons was used to assess the effect of treatment on D-dimer release. Correlations were assessed using Spearman's rank correlation coefficient. To calculate the cut-off for the NETs, a maximally selected rank statistic test was used (maxstat R package version 0.7.26) [17].

All protein measurements were performed in triplicates per sample.

Samples with missing data were excluded from the analyses. For all analyses statistical significance was set at a p-value or adjusted p-value of <0.05.

## Results

### Pleural fluid NET levels in pleural infection and controls

**FIGURE 1:**
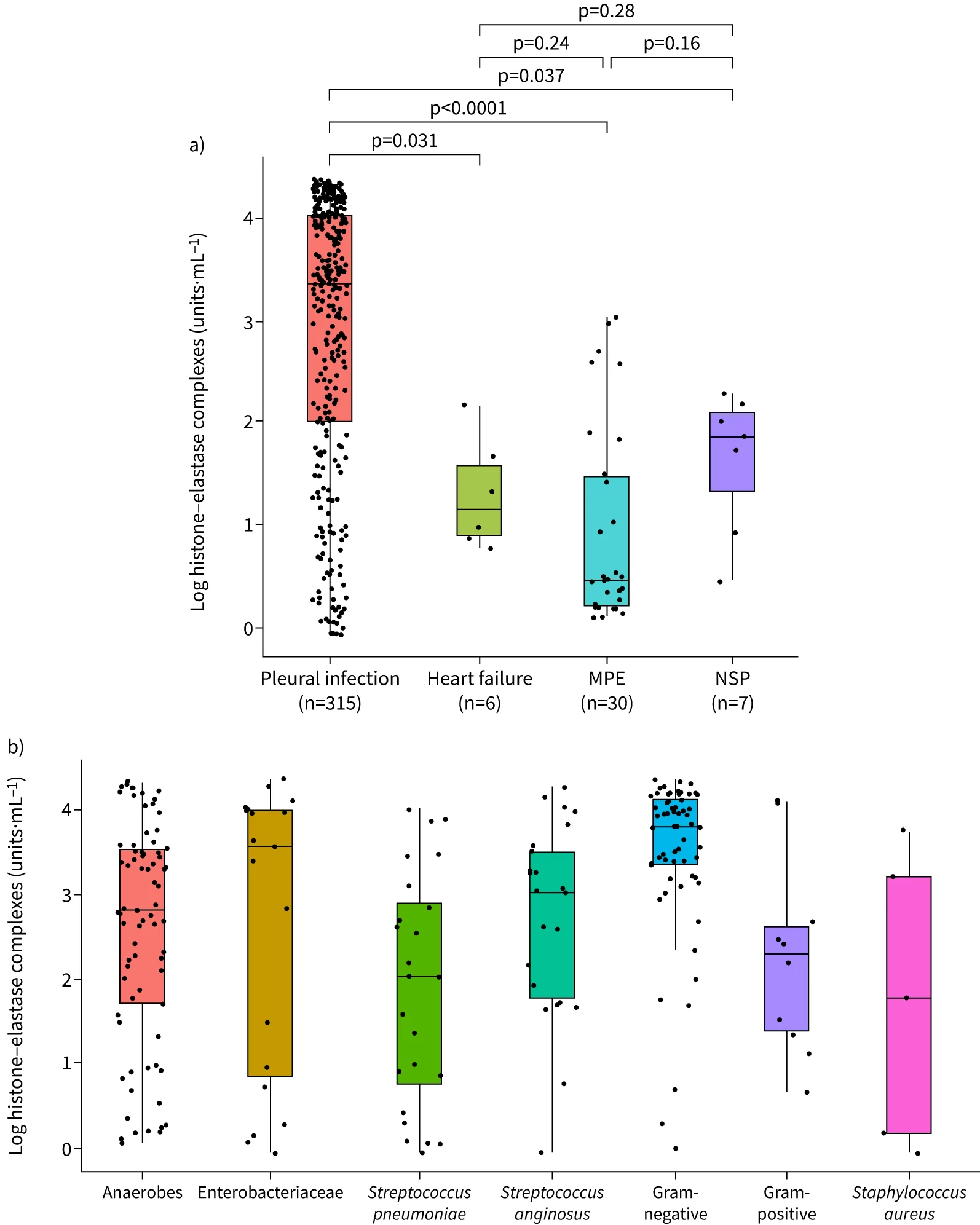
Pleural fluid neutrophil extracellular traps (NETs) are elevated in patients with pleural infection and show an association with the microbiology. a) Pleural fluid NET concentration for patients with pleural disease. Elevated levels were observed for patients with pleural infection (Kruskal–Wallis p<0.0001). The figure presents the adjusted p-values. b) Pleural fluid NET concentration for patients with pleural infection according to microbiology as identified by 16S rRNA next-generation sequencing. Microbiological profiles with Gram-negative bacteria as the most abundant taxa exhibited significantly higher levels of pleural fluid NETs (Kruskal–Wallis p<0.0001). The adjusted p-values are presented in supplementary table S2. Box plots represent median and interquartile range (IQR); whiskers indicate 1.5×IQR. MPE: malignant pleural effusion; NSP: non-specific pleuritis.

The TORPIDS-2 study showed that patients with pleural infection exhibit diverse levels of neutrophil-related immunity activity [7]. To investigate the biological role of NETs in pleural infection we first compared NET levels in the pleural fluid of patients with pleural infection (n=315), heart failure (n=6), malignant pleural effusion (n=30) and non-specific pleuritis (n=7). Demographic characteristics of the study cohorts are summarised in table 1 for the pleural infection group and in supplementary table S1 for the negative control group. Supplementary figure S1 summarises the cohorts that were used for each analysis. Pleural fluid NETs in pleural infection patients exhibited the highest concentration compared with those with malignant pleural effusion (p<0.0001), heart failure (p=0.031) and non-specific pleuritis (p=0.037) (figure 1a).

### Association between pleural fluid NETs and microbiology

We investigated the association between pleural fluid NET concentration and microbiological profiles using the PILOT [13] cohort, for which we have previously performed in-depth molecular microbiology analysis (16S rRNA next-generation sequencing) [15]. Pleural fluid NETs exhibited significant differences according to infecting pathogen (Kruskal–Wallis p<0.0001) (figure 1b and supplementary table S2). Patients with Gram-negative bacteria as the most abundant taxa exhibited the highest mean±sd level of intrapleural NETs (42.94±21.68 units·mL^−1^), followed by those with Enterobacteriaceae (32.49±27.64 units·mL^−1^), strictly anaerobes (24.97±22.78 units·mL^−1^) and *Streptococcus anginosus* Group (24.35±21.32 units·mL^−1^). In contrast, profiles dominated by *Streptococcus pneumoniae* showed the lowest mean±sd levels of pleural fluid NETs (14.34±16.78 units·mL^−1^).

### NET concentration and radiological markers

Patients (PILOT cohort, n=164) presenting with sonographic septations, as assessed by thoracic ultrasound, had significantly higher mean±sd concentrations of pleural fluid NETs (31.05±24.06 units·mL^−1^) compared to those without (22.53±22.44 units·mL^−1^; p=0.02) (supplementary figure S2). A 10-unit increase in pleural fluid NETs was significantly associated with a 16% higher likelihood of sonographic septations (OR 1.17, 95% CI 1.01–1.36; p=0.04). However, no significant association was detected between pleural fluid NETs and the severity of sonographic septations (PILOT cohort, n=164; p=0.2) (supplementary figure S3). These findings combined suggest that pleural fluid NET concentrations are associated with the microbiological causative agent and are associated with the formation of pleural septations in patients with pleural infection.

### NET concentration and clinical risk

**FIGURE 2:**
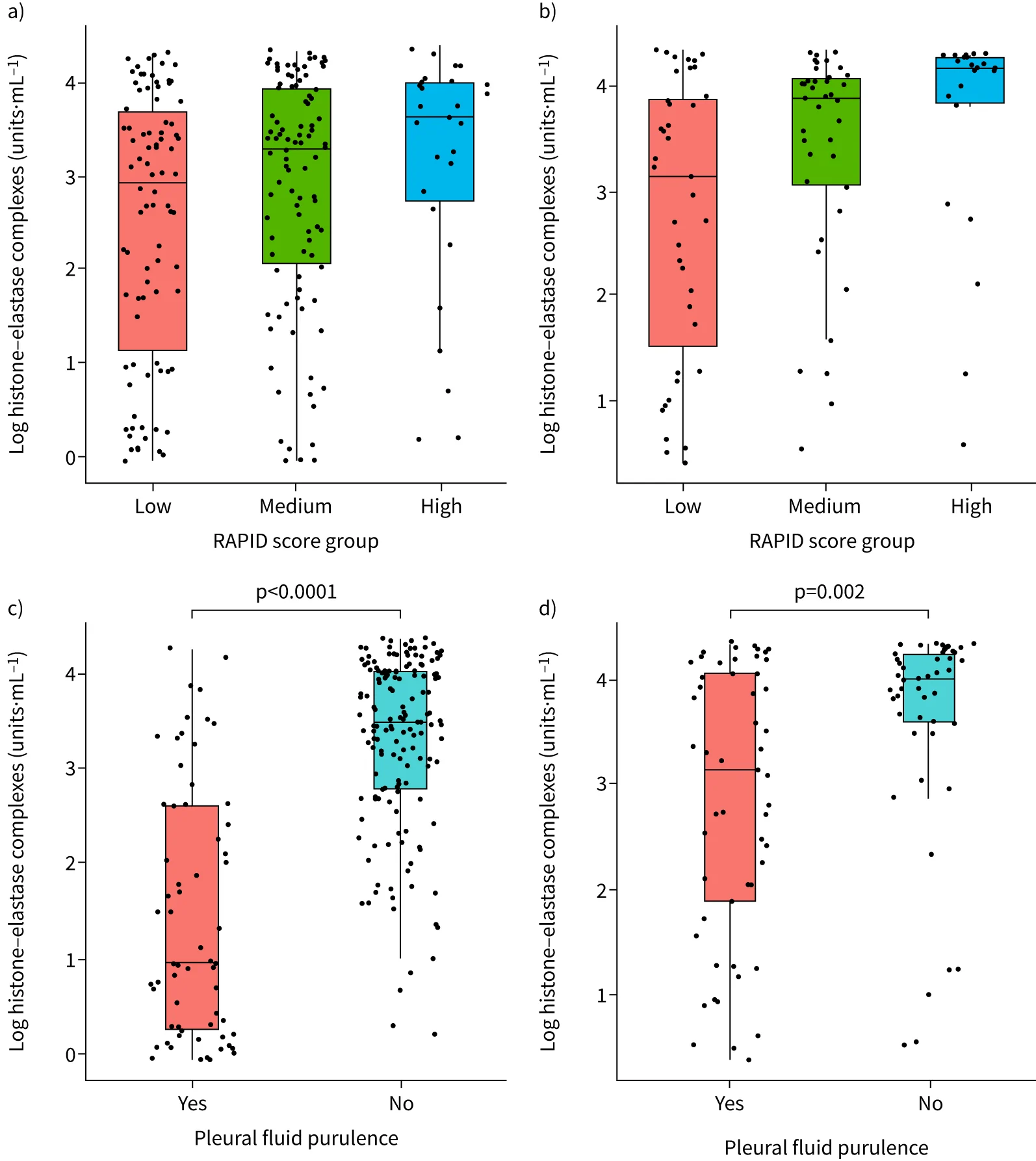
The concentration of pleural fluid neutrophil extracellular traps (NETs) is associated with the severity of disease. a, b) Pleural infection patients exhibited elevating concentrations of pleural fluid NETs as the RAPID score increased: a) OR 1.13, 95% CI 1.02–1.27, per 10 NET units·mL^−1^ (p=0.02) for the PILOT cohort and b) the association was validated on an external and independent cohort (Oxford Pleural Biobank, Oxford-Osler, Patras Pleural Biobank and Ancona Pleural Biobank: OR 1.29, 95% CI 1.11–1.40, per 10 NET units·mL^−1^; p=0.0005). c, d) Patients with purulent pleural fluid exhibited significantly lower levels of pleural fluid NETs: c) p<0.0001 for the PILOT cohort and d) p=0.002 for the external validation cohort. Box plots represent median and interquartile range (IQR); whiskers indicate 1.5×IQR.

We examined the association between the concentration of pleural fluid NETs and the severity of disease as assessed by the RAPID score, which is the only prospectively validated tool that reliably predicts mortality in pleural infection [14]. For the PILOT cohort (n=215) an ordinal logistic regression model demonstrated that a 10-unit increase in pleural fluid NETs was associated with a 13% greater likelihood of belonging to a higher RAPID score group (OR 1.13, 95% CI 1.02–1.27; p=0.02) (figure 2a). Patients in the low RAPID group exhibited the lowest mean±sd (24.98±23.16 units·mL^−1^) pleural fluid NET concentration, followed by patients in the medium (30.23±24.22 units·mL^−1^) and high (36.56±24.12 units·mL^−1^) RAPID groups. No evidence of collinearity was detected between pleural fluid NETs and the RAPID score.

We sought to validate the association between pleural fluid NETs and the RAPID score by combining four cohorts (n=100) which were external and independent of PILOT: Oxford Pleural Biobank, Oxford-Osler, Patras Pleural Biobank and Ancona Pleural Biobank. The analysis confirmed the positive ordinal association between pleural fluid NETs and the RAPID score (OR 1.29, 95% CI 1.11–1.40, per 10 NET units·mL^−1^; p=0.0005) (figure 2b).

Given the association between the RAPID score and NET concentration, we investigated the PILOT dataset (n=215) to determine whether this relationship holds for any of the individual parameters of the RAPID score. No correlation was detected between pleural fluid NETs and age (p=0.7) or urea (p=0.1). However, a weak positive correlation was observed for serum albumin (r=0.18, p=0.007). Patients with purulent pleural fluid exhibited lower mean±sd levels (10.60±15.65 units·mL^−1^) of pleural NETs compared to those without purulence (38.68±22.70 units·mL^−1^; p<0.0001) (figure 2c). This difference was independently confirmed in the validation cohort (n=100; p=0.002) (figure 2d). Logistic regression modelling identified that for each 10-unit increase of pleural NETs the likelihood of a non-purulent effusion increased by 2.08 (OR 2.08, 95% CI 1.64–2.63; p<0.0001) in the PILOT cohort and 1.30 (OR 1.30, 95% CI 1.11–1.52; p=0.001) in the validation cohort. These data taken together demonstrate that pleural fluid NET concentration is associated with the RAPID score and this relationship is dependent on purulence.

### NET concentration and mortality

Subsequently, we sought to investigate whether there was an association between pleural fluid NETs and 1-year survival. A univariate Cox proportional hazards regression analysis stratifying for the RAPID score (PILOT cohort, n=215) revealed that a 10-unit increase in the levels of intrapleural NETs was positively associated with a 15% higher risk of mortality (hazard ratio (HR) 1.15, 95% CI 1.01–1.32; p=0.04). Moreover, a multivariate Cox regression (PILOT cohort, n=152) including pleural fluid NETs, age, sex, peripheral white blood cell count and pleural fluid lactate dehydrogenase demonstrated that only age (HR 1.06, 95% CI 1.03–1.10; p=0.0002) and pleural fluid NETs (HR 1.23, 95% CI 1.02–1.50; p=0.03) remained independently associated with 1-year survival (supplementary table S3).

**FIGURE 3:**
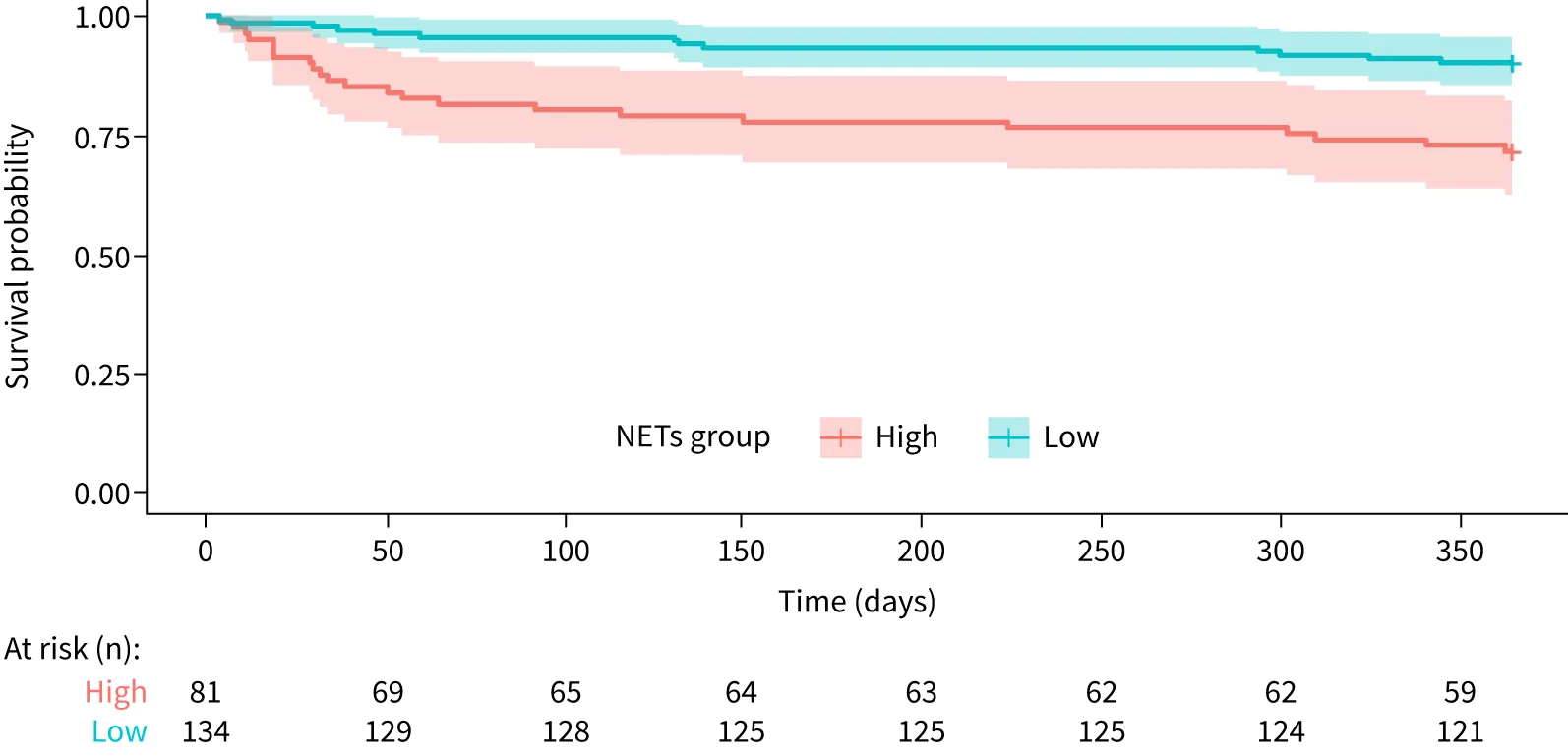
Pleural infection patients with elevated pleural fluid neutrophil extracellular traps (NETs) showed significantly increased risk of mortality. Stratification of patients into high and low NET groups showed that those in the high NET group demonstrated a 3.31-fold higher risk of 1-year mortality (hazard ratio 3.31, 95% CI 1.67–6.54; p=0.0006).

When patients (PILOT cohort, n=215) were stratified into high and low NET groups using a maximally selected rank statistic test, those in the high NET group demonstrated a 3.31-fold higher risk of 1-year mortality (HR 3.31, 95% CI 1.67–6.54; p=0.0006) (figure 3).

Patients in the medium RAPID score group (PILOT cohort, n=98) with high NET levels exhibited a 3.32-fold higher risk of 1-year mortality compared to those with low NET levels (HR 3.32, 95% CI 1.31–8.43; p=0.011) (supplementary figure S4). A similar trend was observed for the high RAPID score group (PILOT cohort, n=27) where patients with high NET levels showed a 1.69-fold higher risk of 1-year mortality. However, this was not statistically significant (HR 1.69, 95% CI 0.52–5.50; p=0.38) (supplementary figure S5). Because only two patients in the low RAPID score group died, this comparison could not be assessed.

To validate the robustness of these findings patients (PILOT cohort, n=215) were also stratified based on the median of pleural fluid NETs. Patients above the median showed a 2.42-fold higher risk of 1-year mortality (HR 2.42, 95% CI 1.19–4.93; p=0.01) (supplementary figure S6).

### NET concentration and need for IET

**FIGURE 4:**
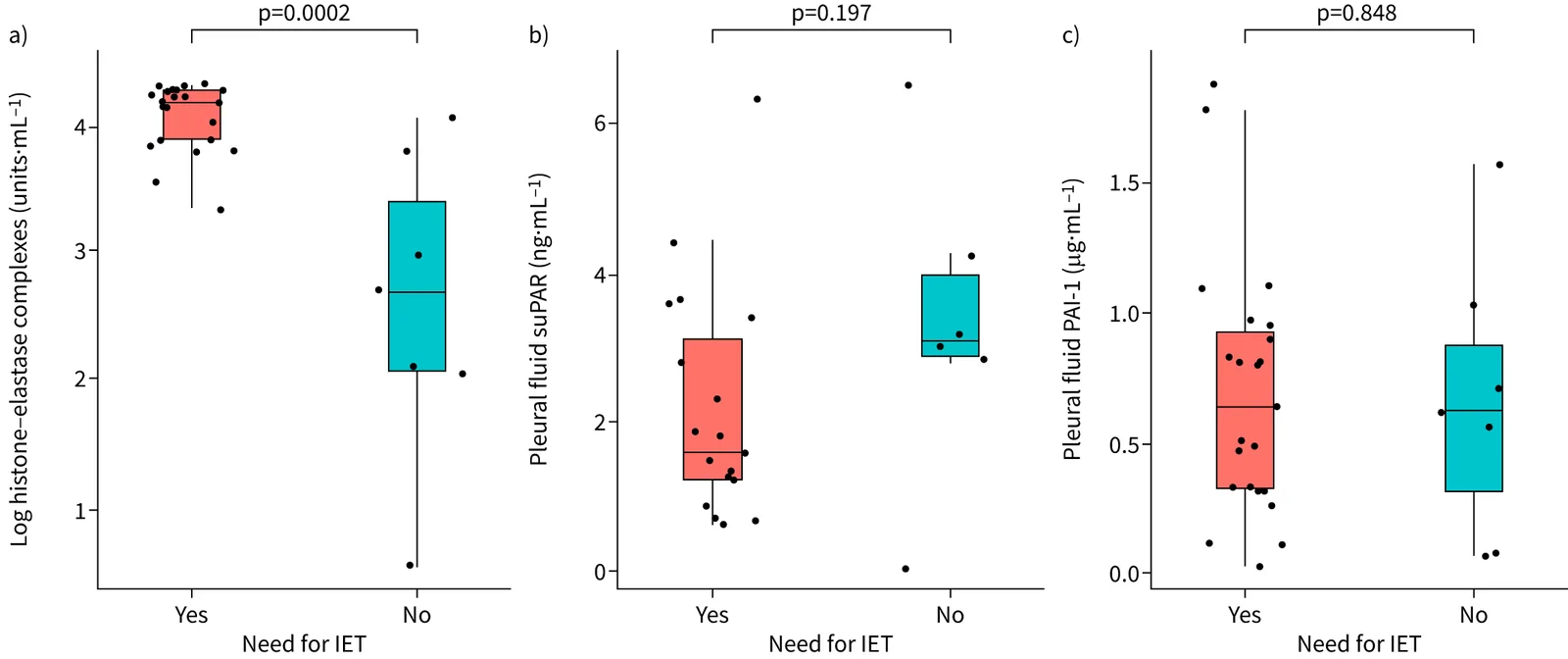
Patients with high concentrations of pleural fluid neutrophil extracellular traps (NETs) were more likely to need intrapleural enzyme therapy (IET). a) Patients who required IET exhibited significantly higher mean±sd levels of pleural fluid NETs (62.10±14.18 units·mL^−1^) compared to those who did not (22.63±21.85 units·mL^−1^; Mann–Whitney U-test p=0.0002). Logistic regression revealed that higher pleural fluid NET concentrations were significantly associated with the need for IET (OR 2.75, 95% CI 1.35–5.62, per 10 NET units; p=0.0055). b, c) No association was detected between need for IET and the mean±sd concentration of pleural fluid b) soluble urokinase plasminogen activator receptor (suPAR) (IET yes: 2.19±1.50 ng·mL^−1^; IET no: 3.31±2.10 ng·mL^−1^; p=0.197) or c) plasminogen activator inhibitor (PAI)-1 (IET yes: 0.69±0.49 μg·mL^−1^; IET no: 0.67±0.53 μg·mL^−1^; p=0.848). Box plots represent median and interquartile range (IQR); whiskers indicate 1.5×IQR.

An exploratory and hypothesis-generating analysis was used to investigate the association between pleural fluid NETs and the need for IET. The criteria to administer IET may vary based on the protocols of each healthcare service and thus for this analysis we used the Oxford-Osler cohort (n=30) as these patients were stratified to treatment following the same clinical guidelines. Mean±sd pleural fluid NET concentrations were significantly higher in patients who required IET (62.10±14.18 units·mL^−1^) compared to those who did not (22.63±21.85 units·mL^−1^; p=0.0002) (figure 4a). Logistic regression highlighted that a 10-unit increase in pleural fluid NET concentrations was significantly associated with a 175% increased odds of requiring IET (OR 2.75, 95% CI 1.35–5.62; p=0.0055). Pleural fluid soluble urokinase plasminogen activator receptor (suPAR) and plasminogen activator inhibitor (PAI)-1 have been suggested to be associated with the need for IET; however, we did not detect significant differences for the Oxford-Osler cohort (p=0.197 for suPAR and p=0.848 for PAI-1) (figure 4b and c).

### Citrullinated fibrin and radiological markers

**FIGURE 5:**
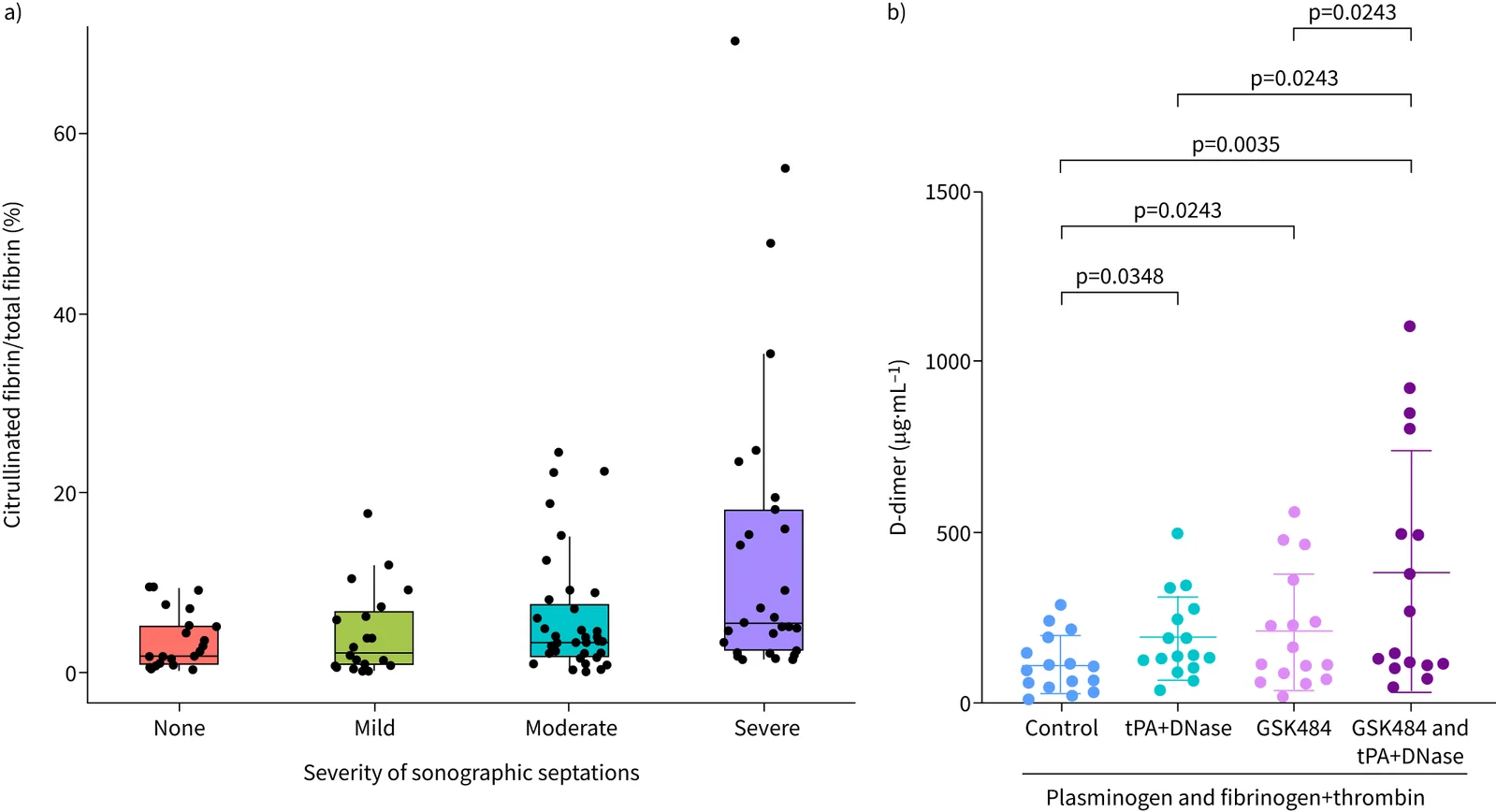
Citrullinated fibrin contributes to the development of sonographic pleural septations. a) A significant positive ordinal association was identified between levels of pleural fluid citrullinated fibrin and the severity of septations (OR 1.10, 95% CI 1.04–1.16; p=0.0005). Box plots represent median and interquartile range; whiskers indicate 1.5×IQR. b) The addition of the peptidylarginine deiminase 4 inhibitor GSK484 to tissue plasminogen activator (tPA) and DNase significantly increased the levels of D-dimer compared to the inhibitor alone (p=0.024) or tPA and DNase (p=0.024). The figure presents the adjusted p-values. Data are presented as mean±sd.

Peptidylarginine deiminase 4 (PAD4) is a key enzyme for the induction of NETosis by mediating histone citrullination, which allows chromatin decondensation. PAD4 released from neutrophils can also citrullinate fibrin, resulting in the formation of denser fibrin clots with decreased porosity that are more resistant to degradation [18], and PAD4 has been detected in pleural fluid from patients with pleural infection [7]. We hypothesised that there may be an association between citrullinated fibrin and sonographic septations. We identified a significant association between levels of pleural fluid citrullinated fibrin and the severity of septations (PILOT cohort, n=107; OR 1.10, 95% CI 1.04–1.16; p=0.0005) (figure 5a). No association was detected between citrullinated fibrin and the RAPID score (p=0.44) (supplementary figure S7) or 1-year survival (p=0.58).

Although citrullination is irreversible and citrullinated fibrin cannot be unmodified, PAD4 inhibitors could block its activity. We investigated *ex vivo* whether the addition of the PAD4 inhibitor GSK484 to tissue plasminogen activator (tPA) and DNase could further enhance fibrinolysis. For this we pre-treated acellular pleural fluid samples from pleural infection patients (n=16) with tPA and DNase or GSK484, or the combination of tPA, DNase and GSK484. The control group was kept untreated. Plasminogen, fibrinogen and thrombin were added to all groups to cause a clot as previously described [19] and the levels of D-dimer were measured. The addition of the PAD4 inhibitor to tPA and DNase significantly increased the levels of D-dimer compared to the inhibitor alone (p=0.024) or tPA and DNase (p=0.003) (figure 5b). Furthermore, consistent results were obtained when two additional PAD4 inhibitors (JBI-589 and JBI-1044) were tested. The combination of PAD4 with tPA and DNase produced the highest increase in D-dimer concentration. In combination, these findings demonstrated citrullination of fibrin by PAD4 contributes to fibrinolytic deficiency in pleural infection patients.

## Discussion

We analysed five independent and prospectively collected cohorts from three countries to investigate the biology of pleural fluid NETs and their associations with microbiology, disease severity, clinical outcomes and clinical phenotypes. To the best of our knowledge, this is the first analysis which specifically demonstrates that pleural fluid NETs associate with microbiological profiles, disease severity, presence of sonographic septations and increased 1-year mortality.

The concentration of pleural fluid NETs varied based on the microbiological profile, with samples dominated by Gram-negative bacteria exhibiting the highest levels (figure 1b). This may be explained by the fact that lipopolysaccharide, which is a key component of the outer membrane of Gram-negative bacteria, plays a central role in the induction of NETosis [20]. It has been suggested that neutrophils can sense the size of microbes and preferentially undergo NETosis in response to larger bacteria which cannot be phagocytosed or when bacteria form large aggregates or biofilms [21]. Samples dominated by *S. pneumoniae*, *Staphylococcus aureus* and other Gram-positive bacteria exhibited the lowest levels of NETs, consistent with existing evidence that these pathogens utilise nucleases to escape from NETs [22, 23].

Our analysis detected a significant ordinal association between pleural fluid NET levels and the RAPID score. Moreover, NETs were positively associated with higher risk of 1-year mortality independently of the RAPID score. However, considering that the RAPID score is a prospectively validated, easy to use and cost-efficient clinical model, further work is needed to evaluate the clinical utility of pleural fluid NETs assessment.

The association between the levels of NETs and the RAPID score was based on purulence, which is one of the parameters of the score [21]. Purulent pleural fluid was classified by macroscopic appearance, reflecting the clinician's contemporaneous assessment of thick, turbid or pus-like effusion at drainage. This pragmatic definition, consistent with international guidelines and prior pleural infection studies [2, 3, 13, 24–26], lacks universally accepted biochemical thresholds. Although this subjectivity may introduce interobserver variability, it reflects real-world decision making. Notably, purulent pleural fluid specimens exhibited lower levels of NETs and this biological finding may explain the now validated clinical observation that purulence is associated with better clinical outcomes in pleural infection [13, 14]. This finding does perhaps challenge assumptions on how purulent material is formed in the pleural space, and we suspect that this is a multifactorial process, with NETs being one contributing factor rather than a sole causal mechanism. Our findings may suggest that increased concentration of NETs reflects more advanced disease; it is established that NETosis is a relatively inefficient and ineffective neutrophil mechanism for bacteria clearance [27].

Biomarkers to inform treatment stratification are currently unavailable and often IET is administered as a rescue treatment, but there is no consensus on the optimal timing of administration. Our exploratory analysis on the Oxford-Osler cohort revealed that patients who required IET exhibited significantly increased levels of pleural fluid NETs. This observation combined with the previous findings suggests a multifaceted role of pleural fluid NETs in pleural infection. Elevated concentrations of NETs may contribute to the stabilisation of fibrin septations, rendering them resistant to clearance by the endogenous intrapleural fibrinolytic activity. Earlier administration of IET in patients with increased pleural fluid NETs may improve clinical outcomes and reduce hospital stay. However, larger studies are required to confirm this hypothesis. A deeper understanding of the factors contributing to the intrapleural fibrinolytic deficiency could lead to more effective and targeted treatments.

The exact pathway underlying septation formation in pleural infection is yet to be discovered. It is thought that bacteria activate the coagulation pathway, leading to the conversion of fibrinogen to fibrin [28]. It has been shown that NETs interact with fibrin to form meshes which are more stable and resistant to degradation [29]. Our findings suggest a further contribution of NETs through PAD4 activity. The levels of citrullinated fibrin were associated with the severity of septations (figure 4a). Notably, in an *ex vivo* fibrin clot model the addition of three different PAD4 inhibitors to tPA and DNase resulted in significantly increased fibrin degradation (figure 4b and supplementary figure S8). These findings require validation in *in vivo* models or clinical studies. Currently, no PAD4 inhibitors have been approved for clinical use [30]; however, should such drugs be developed, future clinical studies may be able to evaluate their fibrinolytic efficacy in patients with pleural infection.

Our study has limitations. While we identified associations and correlations, these do not imply causation. Future studies are necessary to functionally validate these findings. This is a secondary analysis of prospective collected samples and thus the samples were not collected for the primary aim of measuring NETs. We do not have neutrophil counts in pleural fluid to correlate with NETs. Previous work in other diseases has shown that only a subset of neutrophils undergo NETosis and that measurement of NET complexes is more predictive of outcomes than neutrophil counts alone [11, 16], but we are unable to investigate this directly in this study. These findings warrant confirmation in purpose-built cohorts with comprehensive pleural fluid characterisation We analysed pleural fluid specimens which are clinically easily accessible; however, we acknowledge that it remains unclear whether the NETs present in these samples are representative of those found in pleural septations or the pleural tissue *in vivo*. While we measured the concentration of NETs in pleural fluid their functional activity may differ and not directly correlate with concentration levels. The study did not account for specific antibiotic regimens or systemic steroid use, both of which could influence the evolution of pleural infections or potentially the function of neutrophils. Although most of our findings were initially identified in the PILOT [13] cohort and subsequently validated in an international multicohort, the association between pleural fluid NETs and the need for IET was observed in the smaller Oxford-Osler cohort, and thus requires external validation in a larger multicentre cohort.

This study has key strengths including an objective design and a data-driven analysis strategy. We analysed five independent international cohorts of real-life patient samples. All specimens were prospectively collected using consistent inclusion criteria and processed on the day of collection following standardised procedures. Baseline clinical data were available for all samples, and for the PILOT cohort, 1-year survival and microbiology data were also included.

To the best of our knowledge, this is the largest observational study to investigate the biological role of pleural fluid NETs in pleural infection by analysing an international cohort of samples. Our findings demonstrate that NETs play a pivotal role contributing to disease severity, clinical phenotypes and outcomes. In summary, we discovered an association between the underlying microbiology and NET concentration. A 10-unit increase in pleural fluid NETs was associated with greater disease severity, as reflected by higher RAPID scores, an increased risk of 1-year mortality and a higher likelihood of requiring IET. Moreover, NETs may contribute to the development of pleural septations directly through their interaction with fibrin and indirectly through the citrullinating activity of PAD4. These observations open new opportunities to refine pleural infection treatment strategies and raise important questions for future research. For example, do NETs reflect disease progression or resolution during clinical management? Is the accumulation of NETs primarily driven by impaired clearance mechanisms, or does it reflect an increased influx of neutrophils into the pleural cavity? And finally, could therapeutic strategies aimed at inhibiting NETosis improve clinical outcomes in patients with elevated pleural fluid NETs? Significant further work is required to answer these questions. Our data suggest that NET activity and function may be a potential future therapeutic target in pleural infection.

## Data Availability

Anonymised and non-identifiable data included in this study can be made available to other researchers upon request.

## Supplementary material

**Please note:** supplementary material is not edited by the Editorial Office, and is uploaded as it has been supplied by the author. Supplementary material ERJ-00113-2026.Supplement
