# Supplementary material for: Pleural fluid neutrophil extracellular traps are associated with disease severity and risk of 1-year mortality in pleural infection: an observational, international, multicohort study (TORPIDS-3)
Source: Eur Respir J. 2026 Aug 27;68(2):2600113. doi: 10.1183/13993003.00113-2026 (PMC13519835; doi:10.1183/13993003.00113-2026)
Supplement: Supplementary file 1 [file ERJ-00113-2026.Supplement.pdf]

Supplementary Information

Pleural fluid neutrophil extracellular traps are associated with disease severity and risk of one-year mortality in pleural infection. An observational, international, multicohort study (TORPIDS-3)

Julia Y. Chu[1,2], Samuel Hollins[1,2], Alguili Elsheikh[3], Stephen Gerry[4], Nikita Manoharan[1,2], Tryfon Zarganes-Tzitzikas[5,6], Yinzhou Feng[1,2], Elie Antoun[1,7], Eihab O. Bedawi[3], John P. Corcoran[3], Amy Gilmour[8], Federico Mei[9,10], Federica Monaco[11], Marco Tomasetti[11], Argyrios Tzouvelekis[12,13], Electra Koulousousa[12], Fotios Sampsonas[12], Malvika Bhatnagar[3], Ellice Z. Chen[1,2], Paul E Brennan[5,6], Tao Dong[1,7,14], Ian D. Pavord[15], Yuanlin Song[16], James D. Chalmers[8], Julian C. Knight[1,17,*], Najib M. Rahman[1,3,18,*] and Nikolaos I. Kanellakis[1,2,3,18,*]

Affiliations:

1. Chinese Academy of Medical Sciences Oxford Institute, Nuffield Department of Medicine, University of Oxford, OX3 7BN, United Kingdom
2. Laboratory of Respiratory Translational Research, Nuffield Department of Medicine, University of Oxford, Oxford, OX3 7BN, United Kingdom
3. Oxford Centre for Respiratory Medicine, Churchill Hospital, Oxford University Hospitals NHS Foundation Trust, Oxford, OX3 7LE, United Kingdom
4. Centre for Statistics in Medicine, Nuffield Department of Orthopaedics, Rheumatology and Musculoskeletal Sciences, University of Oxford, Oxford, OX3 7LD, United Kingdom
5. Centre for Medicines Discovery, Nuffield Department of Medicine Research Building, University of Oxford, Old Road Campus, Headington, Oxford OX3 7FZ, UK

6. Alzheimer's Research UK Oxford Drug Discovery Institute, Centre for Medicines Discovery, Nuffield Department of Medicine Research Building, University of Oxford, Old Road Campus, Headington, Oxford OX3 7FZ, UK.
7. Centre for Translational Immunology, Nuffield Department of Medicine, University of Oxford; Oxford, OX3 7BN, U.K
8. Division of Molecular and Clinical Medicine, University of Dundee, Ninewells Hospital and Medical School, Dundee, UK
9. Respiratory Disease Unit, Department of Internal Medicine, University Hospital, Ancona, Italy
10. Department of Biomedical Sciences and Public Health, Polytechnic University of Marche, Ancona, Italy
11. Occupational Medicine, Department of Clinical and Molecular Sciences, Polytechnic University of Marche, Italy
12. Respiratory Department, Patras University Hospital, University of Patras, 26500, Patras, Greece
13. Pulmonary Critical Care and Sleep Medicine Department, Yale School of Medicine, USA
14. MRC Translational Immune Discovery Unit, MRC Weatherall Institute of Molecular Medicine, Radcliffe Department of Medicine, University of Oxford, Oxford, OX3 9DS, UK
15. Respiratory Medicine Unit and Oxford NIHR Biomedical Research Centre, University of Oxford, Oxford, UK
16. Shanghai Key Laboratory of Lung Inflammation and Injury, Shanghai Respiratory Research Institute, Department of Pulmonary Medicine, Zhongshan Hospital, Fudan University, Shanghai, 200032, China

17. Centre for Human Genetics, Nuffield Department of Medicine, University of Oxford, OX3 7BN, United Kingdom
18. National Institute for Health Research Oxford Biomedical Research Centre, University of Oxford, Oxford, OX3 7LE, United Kingdom

*Equal contributors

Corresponding Author:

Nikolaos I. Kanellakis PhD

Chinese Academy of Medical Sciences Oxford Institute, Nuffield Department of Medicine, University of Oxford, OX3 7BN, United Kingdom

Nikolaos.kanellakis@ndm.ox.ac.uk

Contents

Supplementary Methods 5
Supplementary Tables 8
Supplementary Figures 10

Supplementary Methods

RAPID Score

The RAPID score is a validated risk prediction score to assess three-month mortality at presentation in adult patients with pleural infection.[1, 2] The RAPID score is using urea (serum), age (years), purulence (yes/no), infection source (community, hospital), and serum albumin. Patients are stratified into three risk groups: low, medium, and high.

TORPIDS-1 Study

The TORPIDS study[3] investigated the microbiology of pleural infection and its association with clinically important outcomes (1-year survival, need for surgery). The microbiology of 243 pleural fluid specimens prospectively collected for the PILOT study was assessed with 16S rRNA next generation sequencing.

TORPIDS-2 Study

The TORPIDS-2 study[4] investigated the host response to pleural infection. Patients with pleural infection exhibited diverse responses of neutrophil-mediated immunity, glycolysis and pentose phosphate activation, which are associated with microbiology.

Inclusion and exclusion criteria for the "Prospective validation of the RAPID clinical risk prediction score in adult patients with pleural infection: the PILOT study" clinical trial[1]

Patients were included if they had a clinical presentation consistent with pleural infection as stated in the Methods section of the main manuscript and any of the following criteria:

1. Pleural fluid that was macroscopically purulent, OR
2. Pleural fluid that was positive on culture for bacterial infection, OR
3. Pleural fluid that demonstrated bacteria on Gram staining, OR
4. Pleural fluid with pH ≤7.2 (measured in a blood gas analyser) or low glucose level (≤3mmol/L
1. or ≤55mg/dL) in a patient with clinical evidence of infection, OR
5. Contrast-enhanced CT evidence of pleural infection (consolidation of underlying lung with enhancing pleural collection) in a patient with clinical evidence of infection, alongside exclusion of other sources of infection.

Exclusion criteria for the PILOT study:

1. Age less than 18 years, OR
2. No pleural fluid available for analysis, OR
3. Patient previous pneumonectomy on the side of pleural infection, OR
4. Expected survival less than three months due to co-morbid disease, as judged by the recruiting physician.

Enzyme-linked immunosorbent assay (ELISA) and Multiplex immunoassay measurement of proteins

A commercial ELISA kit and a multiplex immunoassay kit was used to measure suPAR (E-EL-H2584-ELA, Elabscience) and PAI-1 (LXSAHM-01, R&D System) respectively following manufacturer's instructions.

Measurement of soluble fibrin and citrullinated fibrin
A capture ELISA was developed to quantify soluble fibrin and citrullinated fibrin in pleural fluid samples. All incubation steps in the assay were performed at room temperature and with gentle shaking (300rpm) unless otherwise stated. For quantification of soluble fibrin, a 96 well microplate was coated with undiluted pleural fluid samples overnight at 4°C. After three washes with washing buffer (DY008B, R&D System), wells were blocked with reagent diluent (DY008B, R&D System) for 1 hour, followed by a further three washes. Anti-fibrin (MABS2155, Merck Life Sciences) was added to the plate and incubated for 2 hours. After three washes, the plate was incubated with HRP-conjugated anti-mouse IgG antibody (SA00001-1, Proteintech) for 1 hour and washed three times prior to addition of TMB solution (DY008B, R&D System). Stop solution (DY008B, R&D System) was added to stop reaction after 15-20 minutes of incubation in dark. Absorbance at 450nm and 570nm were measured using a microplate reader (CLARIOstar, BMG Labtech). Quantification of citrullinated fibrin followed the same procedure, except an anti-citrulline antibody (ab100932, Abcam) was used as the capture antibody to coat the microplate overnight, and an extra 2-hour incubation with pleural fluid samples was performed following blocking. The level of citrullinated fibrin was expressed as percentage over total soluble fibrin.

Ultrasound septation score
Objective ultrasound septation s[5]core was assessed by a physician holding Royal College of Radiology Thoracic Ultrasound level 1 competency equivalent or above as previously described.[1, 6]

Measurement of fibrinolysis
Fibrinolysis was measured by performing multiplex immunoassay of D-dimers (LXSAHM-01, R&D System). Pleural fluid samples were pre-incubated with 2µM GSK484 (ab223598, Abcam) alone (or 10µM for JBI-589 and JBI-1044) or in combination of 330pM tPA (T0831, Merck Life Science) and 0.5U Dnase (EN0521, Thermo Scientific) for 1 hour at 37°C prior to addition of 5nM thrombin (HCT-0020, Prolytix) and 0.5mg/ml fibrinogen (HCL-0150R, Prolytix) as previously described.[7] Following a further 4-hour incubation at 37°C, samples were kept at -80°C until use in assay. Assay was performed in a 96 well microplate as per manufacturer's guidelines.

PAD4 Inhibitors JBI-589 and JBI-1044
JBI-589 is a non-covalent, isoform-selective PAD4 inhibitor. In the ammonia release assay, it inhibits human PAD4 with an IC_{50} of 122 nM, while showing no activity against PAD1-3 or PAD6 at concentrations up to 30 µM.[8]

JBI-1044 is a structurally distinct PAD4-selective inhibitor, less characterized chemically but showing strong efficacy in vivo. Preclinical studies report suppression of PAD4-driven pre-metastatic niche formation, resulting in reduced liver metastasis in pancreatic and colorectal cancer models. Beyond oncology, JBI-1044

demonstrates activity in models of fibrosis, colitis, rheumatoid arthritis, and breast cancer.[8]

For the purposes of this study both compounds where synthesised following procedures from their corresponding patents WO2019077631 and WO2022034616.

Supplementary Tables

Supplementary Table 1

**Demographic**	**N**
**Age (years, mean)**	69.6 (14.5)
**Sex**	
Females	20 (47%)
Males	23 (53%)
**Pleural Effusion Aetiology**	
Heart failure	6 (14%)
Malignant pleural effusion	30 (70%)
Non-specific pleuritis	7 (16%)

Supplementary Table 1: The demographic data for the negative control cohort.

Supplementary Table 2

	Anaerobes	***Enterobacteriaceae***	***Streptococcus Pneumoniae***	***Streptococcus Anginosus* Group**	**Gram-negative**	**Gram-positive**
Enterobacteriaceae	1.00	-	-	-	-	-
Streptococcus Pneumoniae	0.35	1.00	-	-	-	-
***Streptococcus Anginosus* Group**	1.00	1.00	0.72	-	-	-
**Gram-negative**	<0.0001	1.00	<0.001	0.02	-	
**Gram-positive**	1.00	1.00	1.00	1.00	0.05	-
Staphylococcus Aureus	1.00	1.00	1.00	1.00	0.18	1.00

Supplementary Table 1: Pleural fluid NETs exhibited significant differences across different bacteriological patterns. The table presents the p values corrected for multiple testing using the Holm method.

Supplementary Table 3

	Hazard Ratio	95% CI	P Value
**Pleural fluid NETs per 10-units**	1.23	1.02-1.50	0.03
**Age**	1.06	1.03-1.10	0.0002
**Sex (male)**	1.023	0.33 - 3.17	0.96
**WBC**	0.99	0.96 - 1.04	0.97
**Pleural fluid LDH**	1.00	0.99 - 1.00	0.85

Supplementary Table 3: A multivariate Cox regression analysis accounting for pleural fluid NETs, age, sex, peripheral white blood cell count, and pleural fluid LDH exhibited that only age and pleural fluid NETs remained independently associated with one-year survival. The table presents the hazard ration, 95% Confidence Interval, and P value for each of the variables.

Supplementary Figures

Supplementary Figure 1

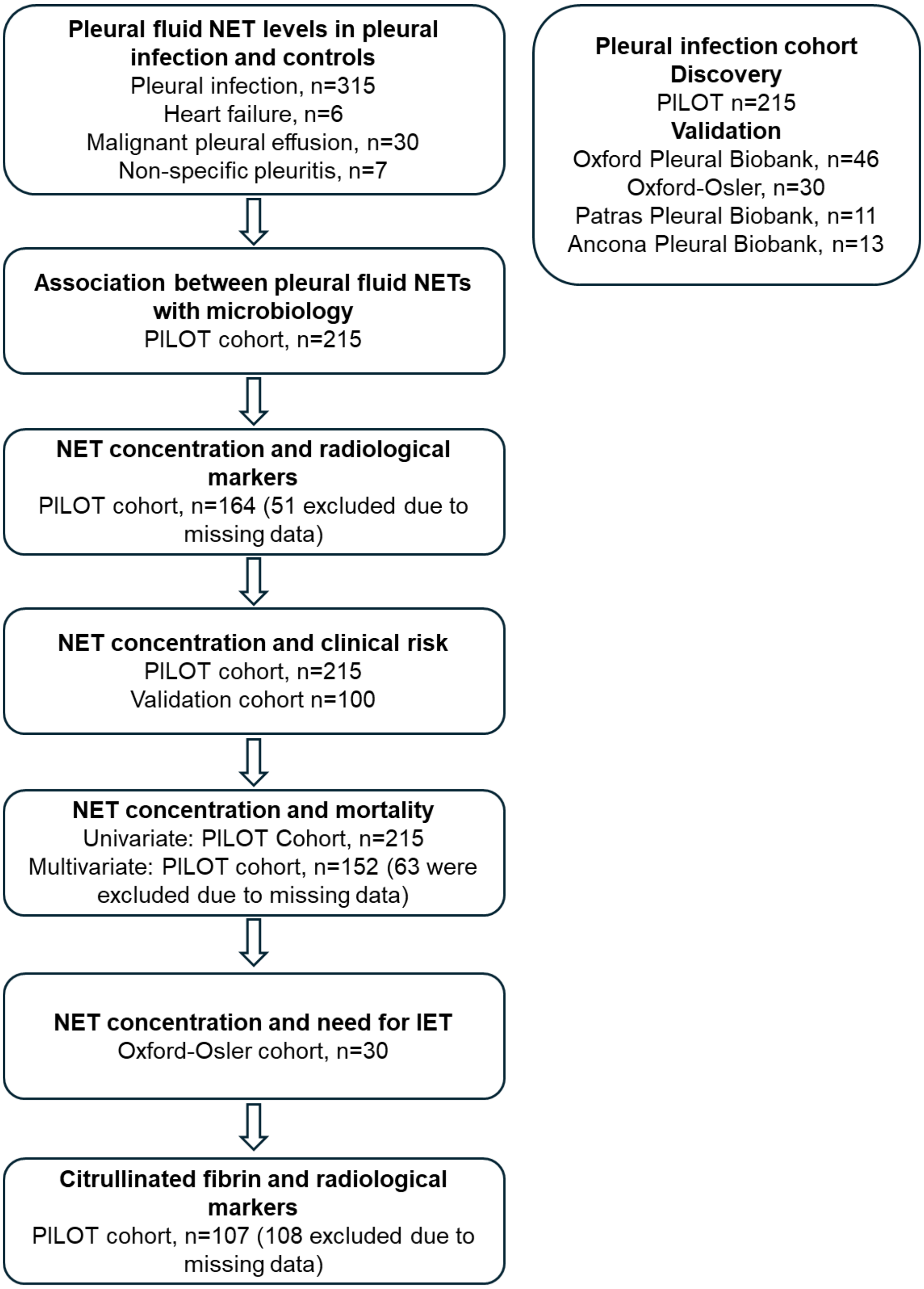

Supplementary Figure 1. Cohorts included in the TORPIDS-3 study. The flowchart presents the cohorts which were used for each analysis, along with their corresponding sample size.

Supplementary Figure 2

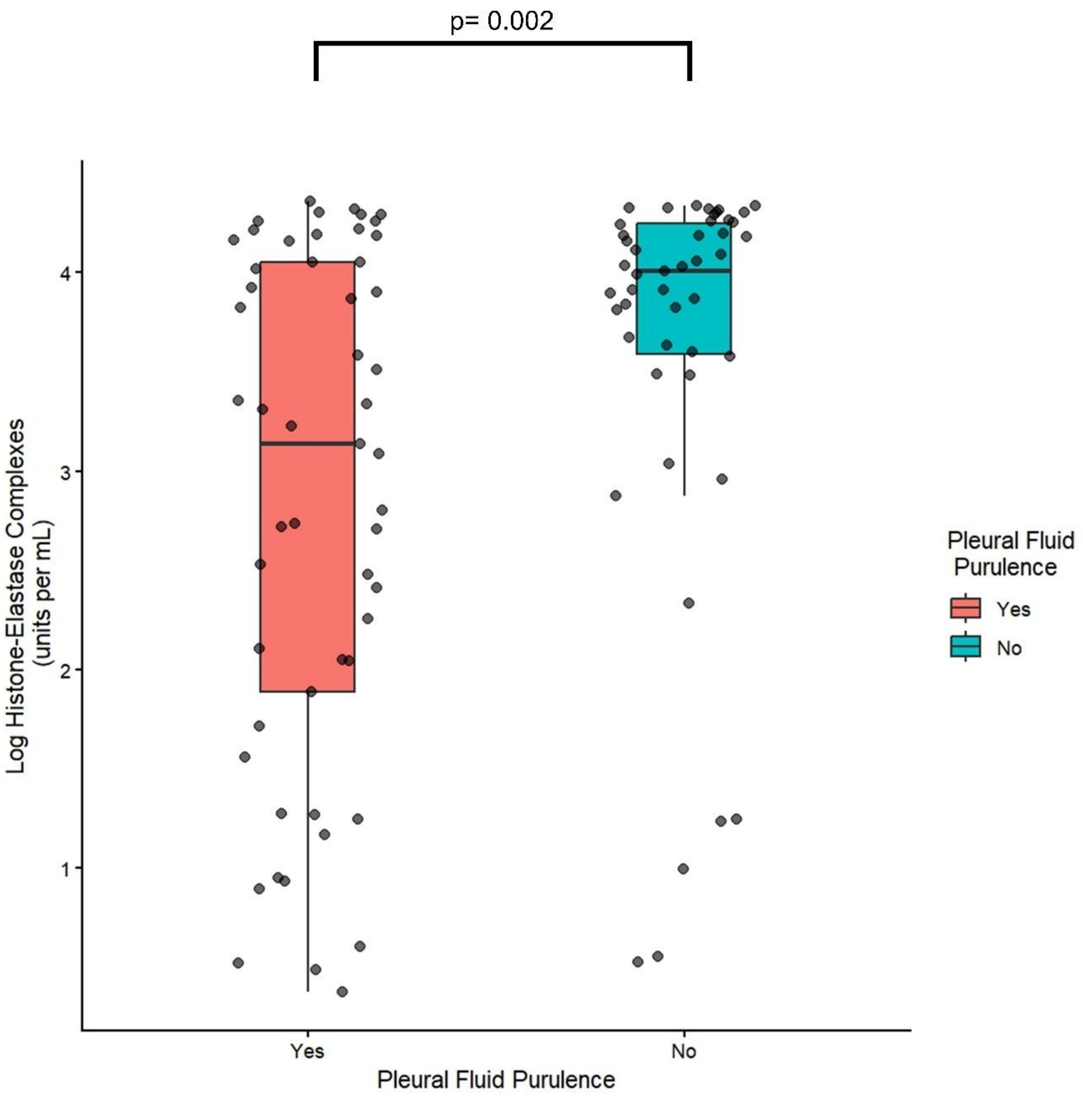

Supplementary Figure 2: Sonographic pleural septations were associated with the level of pleural fluid NETs. Patients presenting with sonographic septations had significantly higher concentrations of pleural fluid NETs (mean=31.05, SD=24.06 units/mL) compared to those without (mean=22.53, SD=22.44, p=0.02, Mann Whitney U test).

Supplementary Figure 3

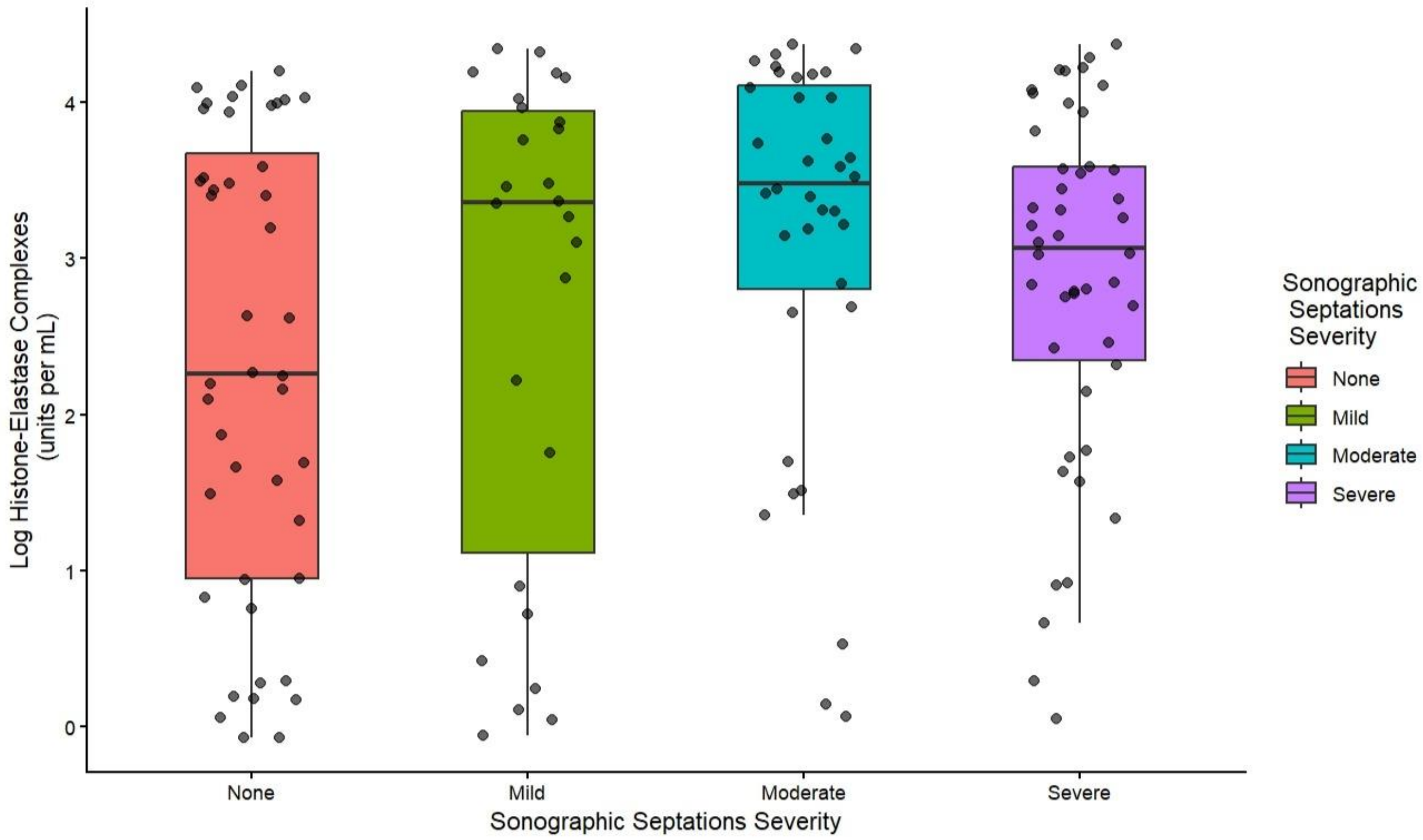

Supplementary Figure 3. Pleural fluid NETs and the severity of sonographic pleural septations. no significant association was detected between pleural fluid NETs and the severity of sonographic septations (p=0.2, ordinal logistic regression).

Supplementary Figure 4

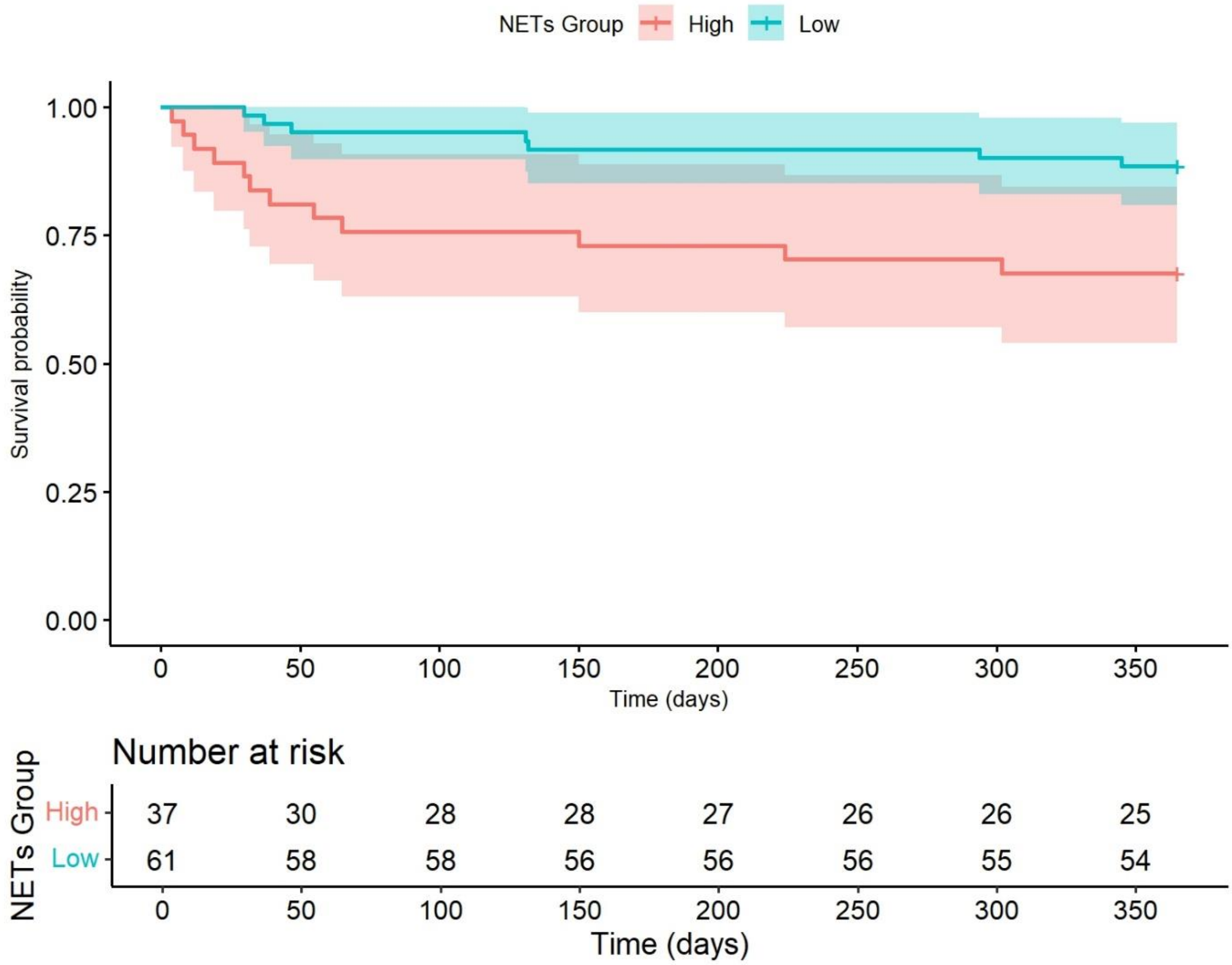

Supplementary Figure 4. Kaplan Meier plot for the Medium RAPID Group. Patients with high NETs concentration exhibited significantly increased risk of one-year mortality (Cox proportional hazards model, HR= 3.32, 95% CI: 1.31-8.43, p=0.011).

Supplementary Figure 5

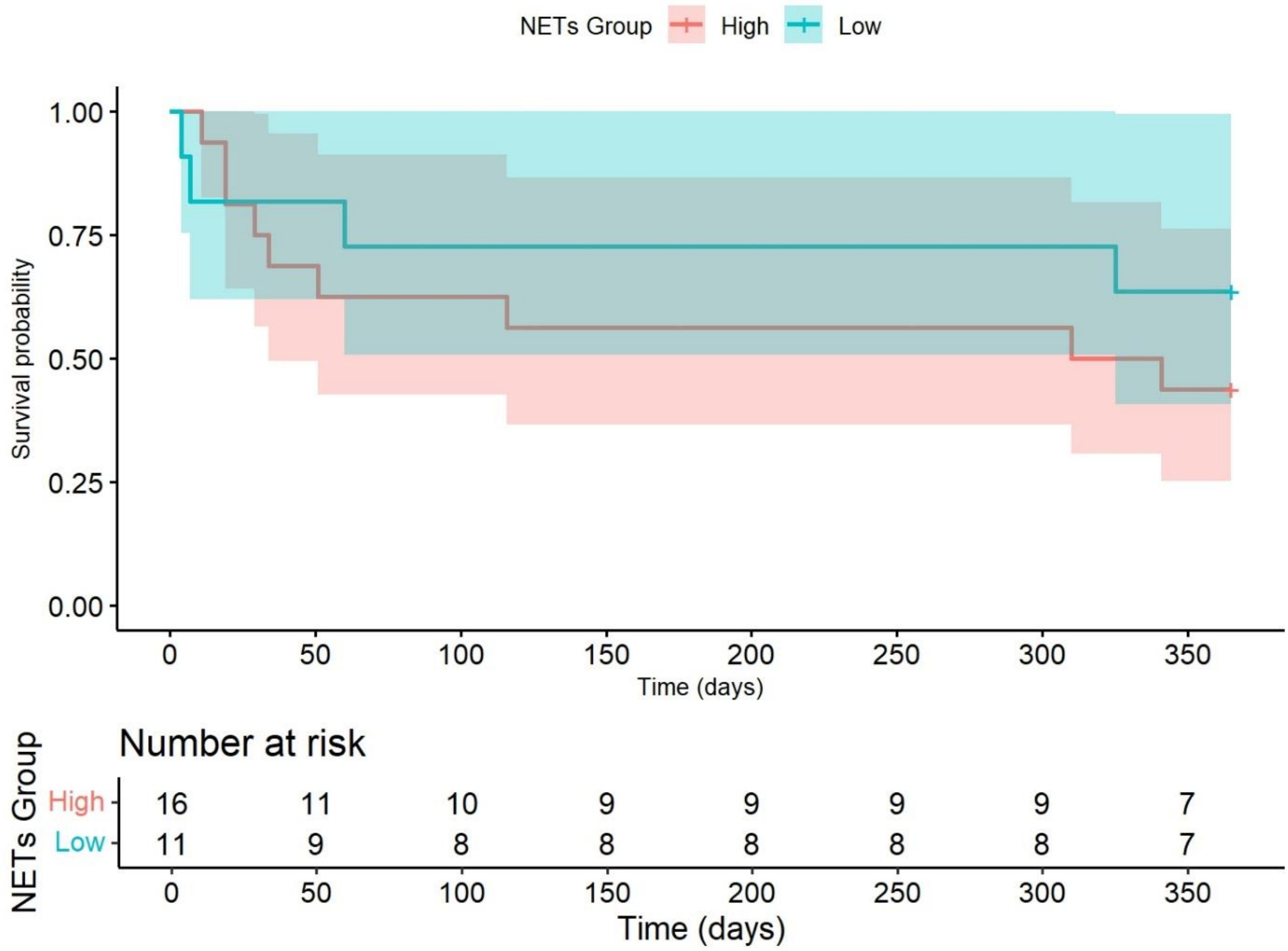

Supplementary Figure 5. Kaplan Meier plot for the High RAPID Group. Patients with high NETs concentration exhibited increased risk of one-year mortality however this was not significant (Cox proportional hazards model, HR=1.69, 95% CI: 0.52-5.50, p=0.38).

Supplementary Figure 6

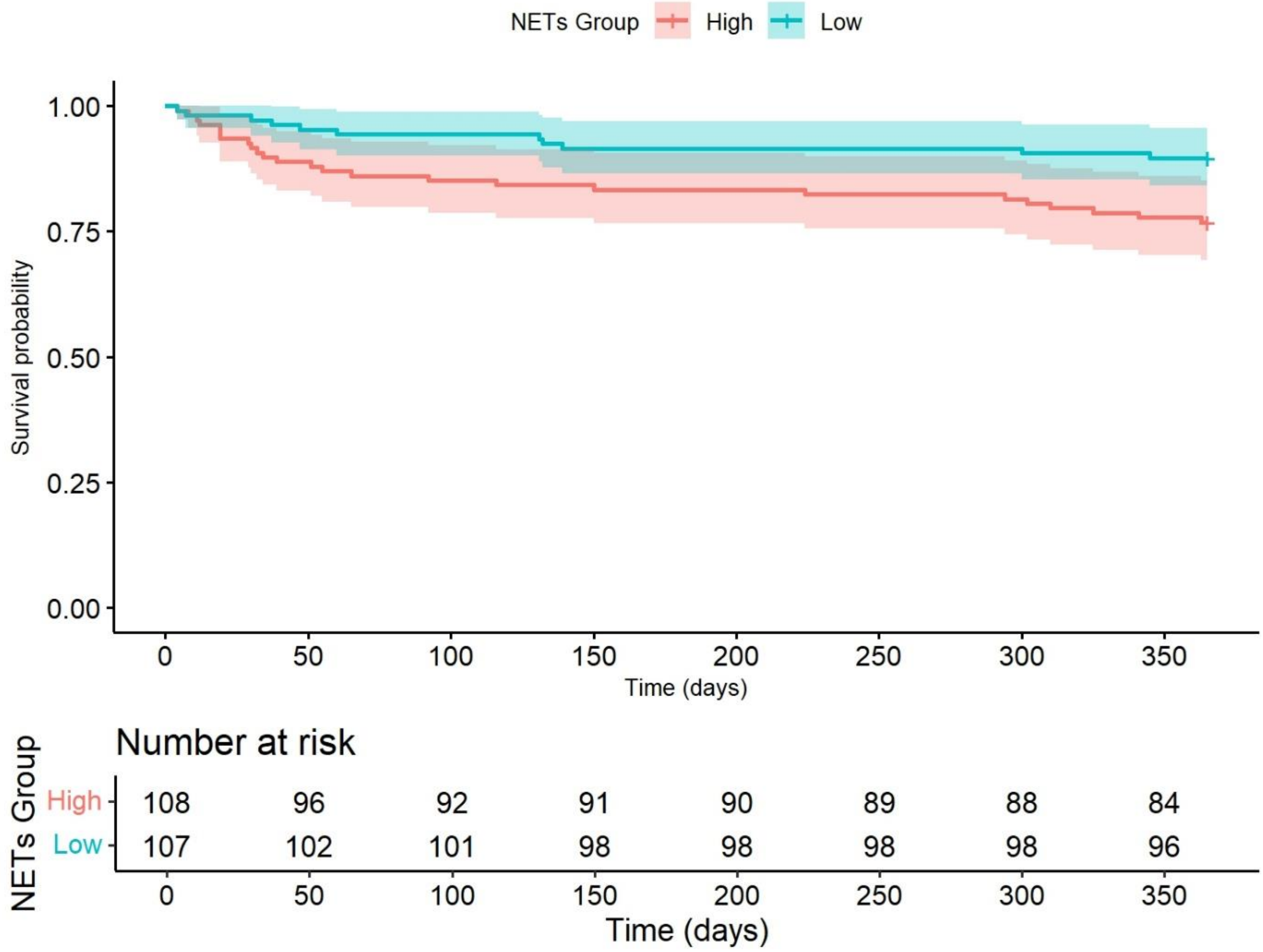

Supplementary Figure 6: The Kaplan-Meier presents the one-year survival for the PILOT cohort (n=215). The patients are separated in the two groups based on whether their pleural fluid NETs are above (high) or below (low) the median of pleural fluid NETs. Patients in the high NETs group showed significantly increased risk of one-year mortality (Cox proportional hazards model, HR= 2.42, 95% CI: 1.19-4.93, p= 0.014).

Supplementary Figure 7

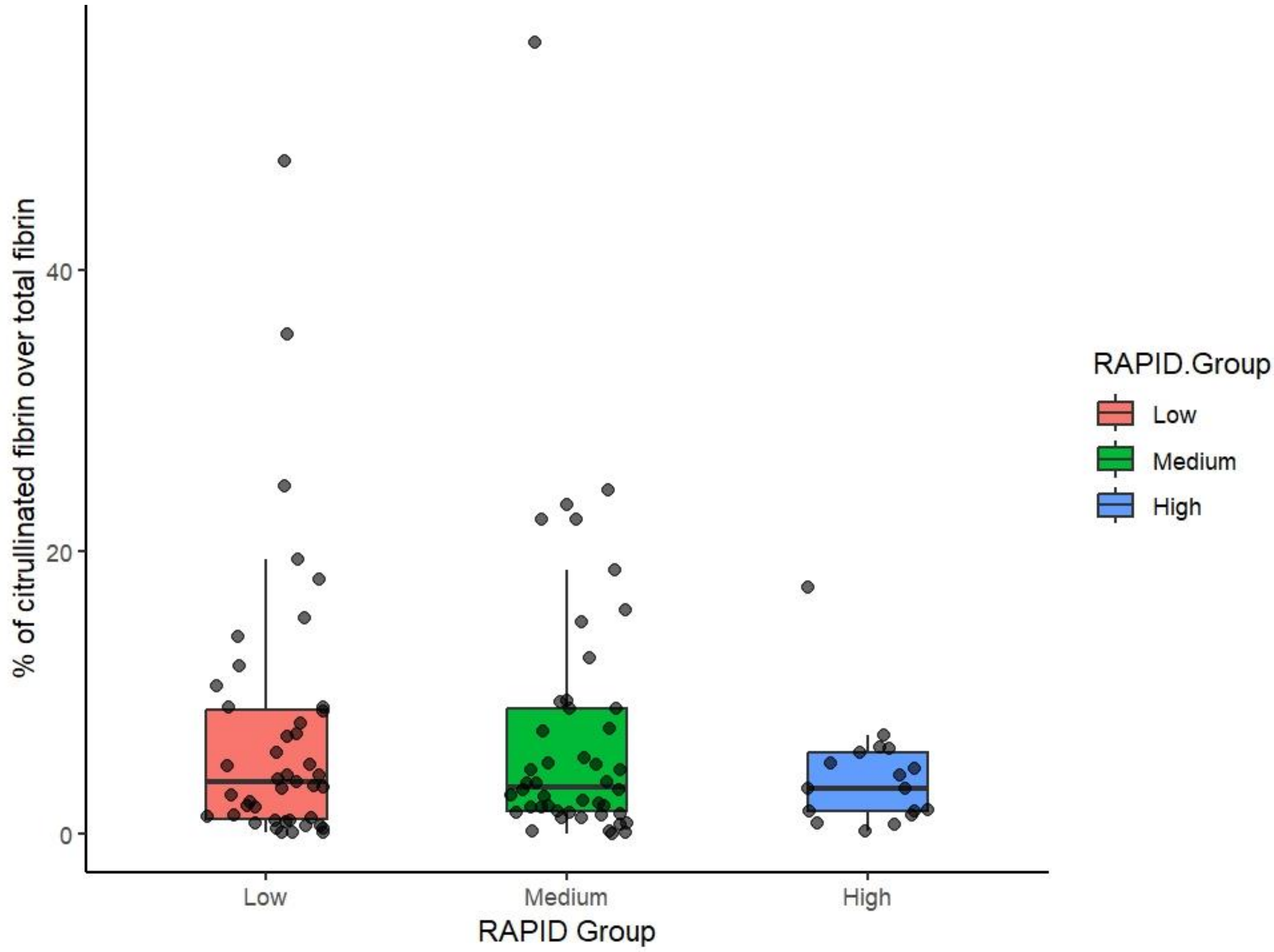

Supplementary Figure 7. No association was detected between citrullinated fibrin and the RAPID score (p=0.44, ordinal logistic regression).

Supplementary Figure 8

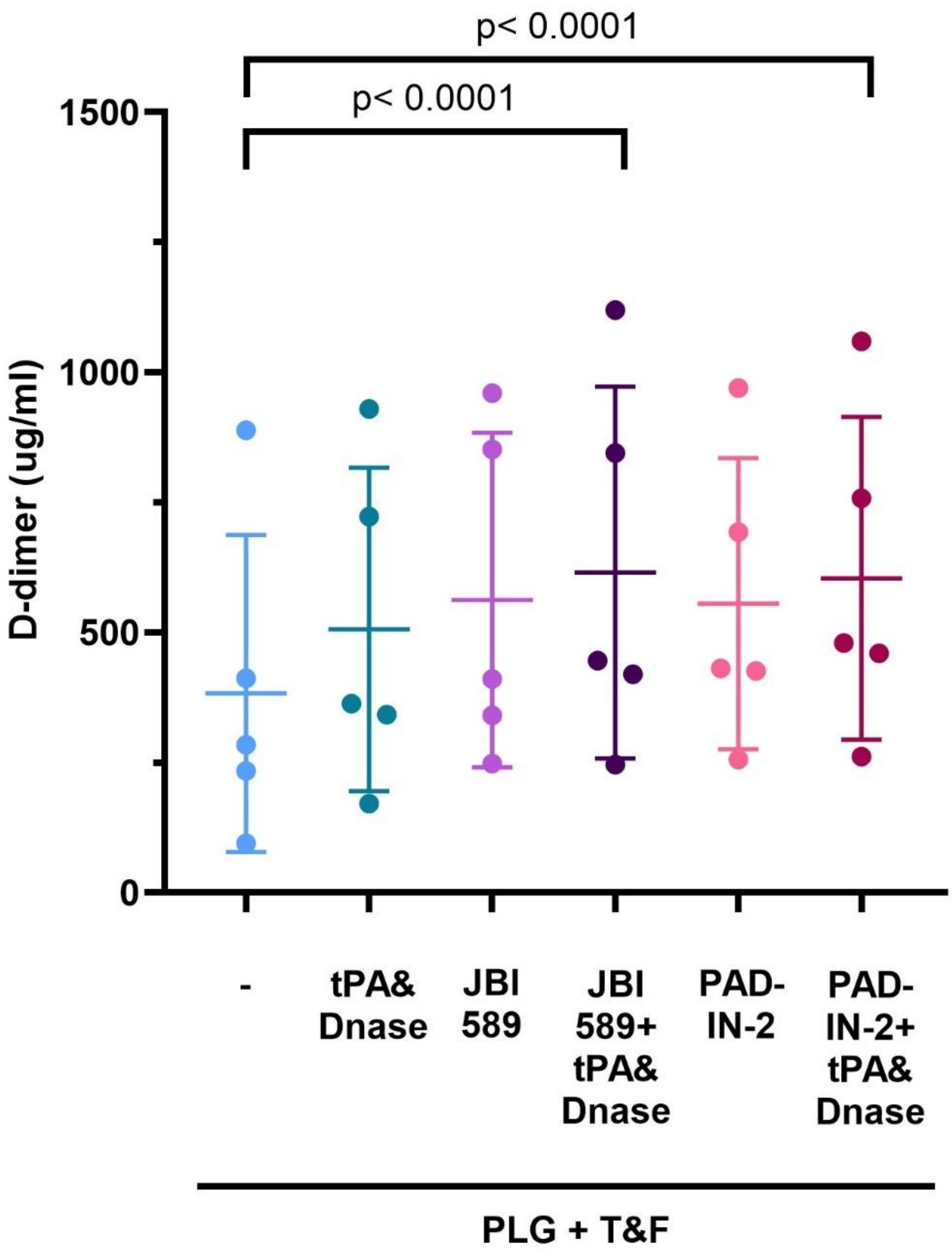

Supplementary Figure 8. The addition of PAD4 inhibitors could enhance fibrinolysis. The combination of two different PAD4 inhibitors (JBI589 and JBI-1044) with tPA and DNAse yielded the highest levels of D-dimers compared to the baseline (p<0.0001).

References

1. Corcoran JP, Psallidas I, Gerry S, Piccolo F, Koegelenberg CF, Saba T, Daneshvar C, Fairbairn I, Heinink R, West A, Stanton AE, Holme J, Kastelik JA, Steer H, Downer NJ, Haris M, Baker EH, Everett CF, Pepperell J, Bewick T, Yarmus L, Maldonado F, Khan B, Hart-Thomas A, Hands G, Warwick G, De Fonseka D, Hassan M, Munavvar M, Guhan A, Shahidi M, Pogson Z, Dowson L, Popowicz ND, Saba J, Ward NR, Hallifax RJ, Dobson M, Shaw R, Hedley EL, Sabia A, Robinson B, Collins GS, Davies HE, Yu LM, Miller RF, Maskell NA, Rahman NM. Prospective validation of the RAPID clinical risk prediction score in adult patients with pleural infection: the PILOT study. *Eur Respir J* 2020: 56(5).
2. Rahman NM, Kahan BC, Miller RF, Gleeson FV, Nunn AJ, Maskell NA. A clinical score (RAPID) to identify those at risk for poor outcome at presentation in patients with pleural infection. *Chest* 2014: 145(4): 848-855.
3. Kanellakis NI, Wrightson JM, Gerry S, Ilott N, Corcoran JP, Bedawi EO, Asciak R, Nezhentsev A, Sundaralingam A, Hallifax RJ, Economides GM, Bland LR, Daly E, Yao X, Maskell NA, Miller RF, Crook DW, Hinks TSC, Dong T, Psallidas I, Rahman NM. The bacteriology of pleural infection (TORPIDS): an exploratory metagenomics analysis through next generation sequencing. *Lancet Microbe* 2022: 3(4): e294-e302.
4. Kanellakis NI, Antoun E, Cano-Gamez K, Chu J, Manoharan N, Berridge G, Vendrell I, Zhang Z, Corcoran JP, Elsheikh A, Dong T, Fischer R, Whalley JP, Knight JC, Rahman NM. Pleural fluid proteomics from patients with pleural infection shows signatures of diverse neutrophilic responses: The Oxford Pleural Infection Endotyping Study (TORPIDS-2). *Eur Respir J* 2025.
5. Keir HR, Shoemark A, Dicker AJ, Perea L, Pollock J, Giam YH, Suarez-Cuartin G, Crichton ML, Lonergan M, Oriano M, Cant E, Einarsson GG, Furrie E, Elborn JS, Fong CJ, Finch S, Rogers GB, Blasi F, Sibila O, Aliberti S, Simpson JL, Huang JTJ, Chalmers JD. Neutrophil extracellular traps, disease severity, and antibiotic response in bronchiectasis: an international, observational, multicohort study. *Lancet Respir Med* 2021: 9(8): 873-884.
6. Bedawi EO, Kanellakis NI, Corcoran JP, Zhao Y, Hassan M, Asciak R, Mercer RM, Sundaralingam A, Addala DN, Miller RF, Dong T, Condliffe AM, Rahman NM. The Biological Role of Pleural Fluid PAI-1 and Sonographic Septations in Pleural Infection: Analysis of a Prospectively Collected Clinical Outcome Study. *Am J Respir Crit Care Med* 2023: 207(6): 731-739.
7. Barrett CD, Moore PK, Moore EE, Moore HB, Chandler JG, Siddiqui H, Maginot ER, Sauaia A, Perez-Calatayud AA, Buesing K, Wang J, Davila-Chapa C, Hershberger D, Douglas I, Pieracci FM, Yaffe MB. Neutrophil-Mediated Inflammatory Plasminogen Degradation, Rather Than High Plasminogen-Activator Inhibitor-1, May Underly Failures and Inefficiencies of Intrapleural Fibrinolysis. *Chest* 2025: 167(1): 67-75.
8. Aiken SG, Grimes T, Munro S, Zarganes-Tzitzikas T, La Thangue NB, Brennan PE. A patent review of peptidylarginine deiminase 4 (PAD4) inhibitors (2014-present). *Expert Opin Ther Pat* 2025: 35(6): 611-621.
